# A Case Report on Subcutaneous Emphysema and Pneumomediastinum Following a Routine Dental Procedure

**DOI:** 10.7759/cureus.41177

**Published:** 2023-06-30

**Authors:** Seth J Deskins, Nancy E Brunner, Matthew Brunner

**Affiliations:** 1 Internal Medicine/Pediatrics, West Virginia University (WVU) Medicine, Morgantown, USA; 2 Pediatrics, West Virginia University (WVU), Morgantown, USA; 3 Internal Medicine/Pediatrics, West Virginia University (WVU), Morgantown, USA

**Keywords:** omfs, diagnosis, intervention, general internal medicine, air, subcutaneous emphysema, dental cavities, dental, causes of pneumomediastinum, pneumomediastinum

## Abstract

Subcutaneous emphysema (SE) and pneumomediastinum are rare complications of air beneath the skin layers and in the mediastinal space, respectively, following routine dental procedures. A few cases exist in the literature. A 53-year-old female presented to the emergency department shortly after a cavity filling, with marked swelling of her right orbit, face, and neck. Physical examination and computed tomography (CT) revealed subcutaneous emphysema and pneumomediastinum. The patient was treated with prophylactic antibiotics for one week and Peridex rinse twice daily. Subcutaneous emphysema and pneumomediastinum cases have been associated with potentially life-threatening sequelae and infections. Although these conditions are almost exclusively benign and self-limiting, physicians should consider the associated fatal complications and manage accordingly. Dental providers should be able to recognize this complication and provide patients with appropriate guidance.

## Introduction

The etiology of subcutaneous emphysema (SE) and pneumomediastinum is broad, and these can occur after trauma to the head and neck and after invasive procedures such as bronchoscopy and have even been seen after the use of wind instruments [1]. Their occurrence is rare, though, after dental procedures [2]. When these complications are seen secondary to dental procedures, they typically are noted to occur during tooth extractions or when a high-speed drill is used [3,4]. Diagnosis is made with history and physical examination that is corroborated with radiography and computed tomography (CT) imaging [5]. Treatment is often supportive with self-resolution. We present an uncommon case of massive cervicofacial subcutaneous emphysema and pneumomediastinum after a routine dental procedure.

## Case presentation

**Figure 1:**
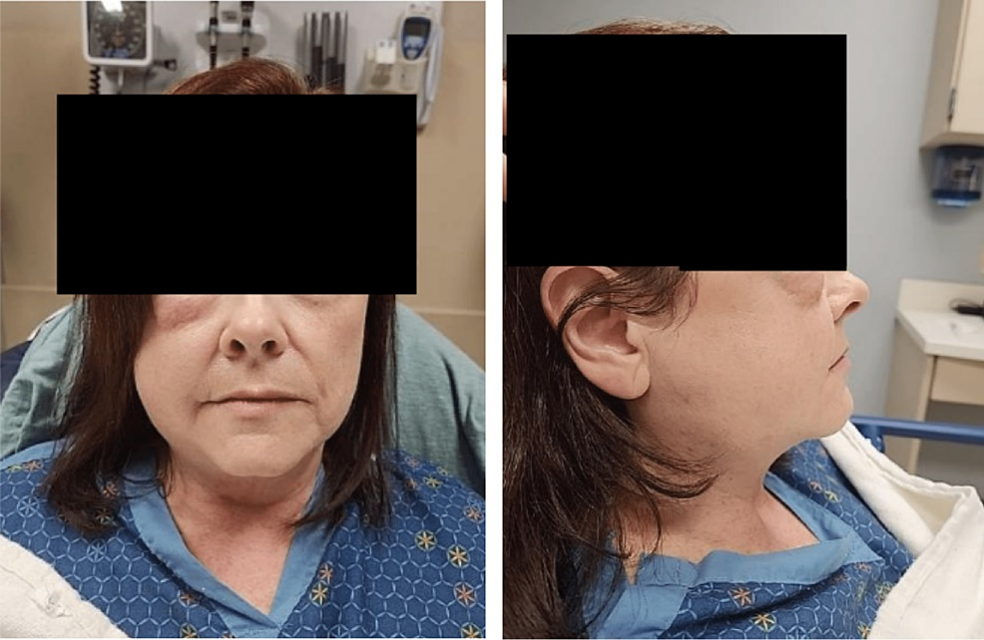
Frontal and lateral profiles with marked swelling of the right orbit, face, and neck

A 53-year-old female with a past medical history of celiac disease, gastroesophageal reflux disease, and hypothyroidism presented to the emergency department after a sudden onset of right-sided facial and eye swelling. She was seen earlier that morning at a local dental office, where she underwent a replacement of a prior filling. Upon presentation, a review of systems was negative except for right-sided blurry vision. She denied headache, nausea, emesis, dyspnea, chest pain, fever, or rash. She had no allergies and received no new medications during the procedure. Vital signs were within normal limits. Physical examination was remarkable for crepitus of the right lateral neck, mandibular, midface, and periorbital area (Figure 1).

**Figure 2:**
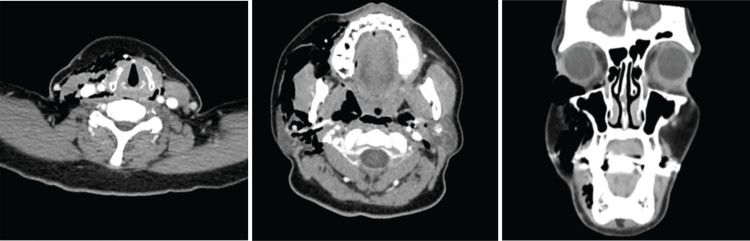
Multiple CT views showing extensive subcutaneous emphysema CT: computed tomography

CT of the soft tissue of the neck and chest was obtained. Imaging revealed extensive pneumomediastinum, the right greater than the left, with an extension into the soft tissues of the neck and adjacent to and within the right orbit without the evidence of pneumothorax, focal inflammation, or abscess (Figure 2).

Oral maxillofacial surgery (OMFS) and thoracic surgery were consulted. OMFS recommended the initiation of prophylactic amoxicillin-potassium clavulanate for a one-week duration along with Peridex rinse twice daily. Thoracic surgery evaluated the patient and agreed with the initiation of prophylactic antibiotics. Neither surgical service recommended surgical intervention. The patient desired to be discharged home with close outpatient follow-up. Return precautions, including worsening swelling, chest pain, dyspnea, and fever, were given to the patient upon discharge.

She was re-evaluated for acute shortness of breath and tachypnea in the emergency department approximately one week after her initial presentation. Her clinical presentation was concerning for pulmonary embolism. Repeat CT of the chest was performed and did not reveal pulmonary embolism but did reveal a vast improvement in subcutaneous emphysema and pneumomediastinum from the week prior without any interventions. She was diagnosed with a panic attack and discharged home.

## Discussion

The development of subcutaneous emphysema (SE) and pneumomediastinum was initially described by Turnbull in 1900 when a musician blew his bugle directly after a tooth extraction [6]. This occurs due to the introduction of air in the subcutaneous space that leads to air being able to track along the fascial planes via the path of least resistance, which typically involves the infraorbital, cervical, and lateral pharyngeal spaces [2]. This, as a result of dental procedures, is very uncommon. Two separate review series looking at cases over the span of approximately 50 years noted just under 150 total cases attributed to dental procedures [1].

The etiology of SE is broad. A major category is trauma to the head and neck [2]. Invasive procedures, such as laparoscopic surgery, bronchoscopy, gastric tube placement, and mechanical ventilation, have also been linked to SE and pneumomediastinum [2]. Other medical conditions to consider are underlying infection and preexisting pulmonary pathology, such as asthma [2]. Some rare cases involving woodwind instruments are also documented as causal for SE. Subcutaneous emphysema can also be idiopathic.

The patients typically present within hours of the inciting procedure. History and physical examination are important for diagnosis. Crepitus on palpation of the neck is pathognomonic for SE [2]. When pneumomediastinum is present, Hamman’s sign, a crunching sound heard with each heartbeat, can be appreciated with auscultation [2]. Radiography and CT of the soft tissue of the neck and chest can confirm the diagnosis and reveal radiolucent layering of air tracking through facial planes. Imaging plays a role in ruling out life-threatening pathologies, such as pneumothorax, air embolism, and cardiac tamponade [2,4]. CT is the most sensitive imaging modality for confirming the diagnosis [3].

Subcutaneous emphysema is largely benign. Most cases have spontaneous resolution within 2-10 days [3]. For patients admitted to the hospital, the use of oxygen can be considered as it hastens resolution, which is attributed to nitrogen washout [3]. Some of the most worrisome sequelae are the development of tension pneumothorax and air embolism, both of which can be life-threatening without urgent intervention [2]. There have also been some reports of blindness due to orbital involvement [2]. If the retropharyngeal space is involved, tracheal compression can occur [2]. A major concern with cervicofacial emphysema is infection. Oral bacteria can be introduced via emphysematous tracts and can travel along fascial planes leading to serious infections such as necrotizing fasciitis or mediastinitis [1,2]. Due to infections, the use of prophylactic antibiotics is the standard of care [1,2]. The use of corticosteroids can be considered, but there is no consensus regarding their routine use [2].

## Conclusions

Dental procedures are very common. Cervicofacial and mediastinal emphysema represent rare complications. Physical examination and history are key to establishing the diagnosis. Physicians should remain cognizant because, although almost exclusively benign, with self-resolution, there are potentially fatal complications to consider, which should be ruled out. Treatment should consist of the initiation of prophylactic antibiotics and the consideration of supplemental oxygen and corticosteroids. We present this case to raise awareness for providers and emphasize the importance of recognizing a very rare complication from a common dental procedure.
